# In Silico Subtractive Proteome Analysis to Design Multi-Epitope-Based Subunit Vaccine against *Eikenella corrodens*

**DOI:** 10.4014/jmb.2410.10015

**Published:** 2024-11-25

**Authors:** Fatemah AlMalki

**Affiliations:** Fatemah AlMalki, Biology Department, College of Science and Humanities- Al Quwaiiyah, Shaqra University, Al Quwaiiyah 19257, Saudi Arabia

**Keywords:** Immunoinformatics, multi-epitope vaccine, *Eikenella corrodens*, penicillin-binding protein 1A (PBP1A), Toll-like receptors (TLRs)

## Abstract

*Eikenella corrodens* is a gram-negative, facultatively anaerobic bacterium typically found in the oropharynx and respiratory tract of humans. It is responsible for various infections, including head-and-neck infections, pericarditis, and abscesses of the deltoid, perirenal tissue, brain, and liver. Increasing antibiotic resistance requires urgent identification of novel drug targets to fight this bacterium. In this study, subtractive proteomics and immunoinformatics approaches were used to identify the most suitable candidates for multi-epitope vaccine development. A non-homologous and pathogenic protein, penicillin-binding protein 1A (PBP1A), was identified after extracting the entire proteome sequence of *E. corrodens* NCTC 10596. PBP1A is antigenic and necessary for pathogen survival. Helper T-cell (HTL), cytotoxic T-cell (CTL), and B-cell lymphocyte-inducing epitopes were integrated through immunoinformatic methods and rigorous immunological screening processes. Various physicochemical, allergenic, and antigenic properties were also evaluated to ensure the safety and immunogenicity of the vaccine candidates. Dynamic modeling and molecular docking techniques were used to examine the molecular interactions, thermodynamic stability, and binding affinities. The vaccine demonstrated a robust and consistent interaction with Toll-like receptors (TLRs), and its potential to elicit an immunological response was evaluated in silico. For in silico cloning, the final vaccine candidates were back-translated and cloned into an *E. coli* host to achieve high expression of the predicted protein. Computational analyses suggested that the proposed vaccine candidate shows promise for combating bacterial infections and eliciting a robust immune response. However, experimental validation is crucial to authenticate the precise safety and immunogenicity profiles of this vaccine.

## Introduction

*Eikenella corrodens* is a gram-negative, facultative anaerobe that causes various infections, including brain abscesses, periodontitis, endocarditis, intra-abdominal infections, osteomyelitis, and pleuropulmonary infections [1]. *Eikenella* spp. have been linked to a range of infections in adults, including infections of the head and neck, sinusitis, lung infections, arthritis, pancreatic abscesses, infections of the skull, spinal osteomyelitis, and infections from wounds caused by human bites [2]. Although most infections are benign and mild, severe and invasive infections can develop under specific circumstances [3].

Penicillin, tetracycline, and chloramphenicol are effective as oral antibiotics, whereas cephalexin, dicloxacillin, and similar antibiotics are generally not used as oral treatments [1]. Third-generation cephalosporins and cefoxitin exhibit potent activity against *Eikenella* [2]. Nevertheless, owing to factors such as genetic mutations, horizontal gene transfer, and inappropriate use of antibiotics, *E. corrodens* and other similar pathogens can ultimately develop resistance to antimicrobial agents [4]. This resistance makes treating illnesses increasingly difficult [5], and therefore it is crucial to identify novel pharmacological targets and develop innovative therapeutic strategies [6]. Recent advances in sequencing technologies have made extensive genomic information available, facilitating the screening of new vaccine targets [7]. Researchers have been able to develop effective vaccines against a variety of diseases using new techniques in subtractive proteomics and immunoinformatics [8].

The conventional method of vaccine development, which uses large proteins or entire organisms, increases the risk of adverse reactions and creates an excessive antigenic burden [9]. To address these concerns, we proposed an epitope-focused vaccination approach [10]. This method uses small epitopes capable of triggering robust and precise immune reactions, while mitigating the risk of allergic responses [10]. The use of subtractive proteomics in conjunction with immunoinformatics to develop efficient, low-cost immunizations against various illnesses has become popular [11], and this approach is now being widely employed to develop vaccine candidates against viral and bacterial infections [12].

In this study, we used in silico approaches to develop dynamic, potent vaccinations to prevent *E. corrodens* infection by linking the *E. corrodens* NCTC 10596 proteome with genetic data to identify possible vaccine targets. Epitopes for T and B cells were predicted using proteins from *E. corrodens*. We designed highly conserved antigenic epitopes to develop subunit vaccines, and adjuvants and linkers were subsequently incorporated. Online resources were used to assess physicochemical attributes, structural characteristics, allergenic potential, and antigenicity of the vaccines. Additionally, docking between the vaccination and TLR-4 was performed, and in silico cloning was used to study advanced polyprotein synthesis.

## Materials and Methods

### Proteome Retrieval

The UniProt database is an essential repository of protein information that includes cross-references to more than 150 new databases. It provides the proteome of *E. corrodens* NCTC 10596 in the FASTA format [13].

### Vaccine Candidate Prioritization

Key proteins are essential for cell existence [14]. Uploading the core proteome of *E. corrodens* strains onto the Geptop 2.0 server enabled the identification of crucial proteins in those strains [15]. A threshold value for essentiality of 0.24 was determined [16]. Any type of bacterium whose genome has been sequenced can be treated with Geptop [15]. The essential proteins were analyzed using BLASTP with default parameters to identify sequences unique to *E. corrodens* by comparing them with the human proteome, focusing on non-homologous proteins [17]. Non-homologous proteins were defined as those that exhibited no similarity to the human proteome [18]. The accurate prediction of subcellular protein localization is crucial for genome annotation and bacterial infection analysis because such proteins may serve as primary targets for medications or vaccines [19]. Subcellular localization of non-homologous proteins was predicted using the PSORTb server [20]. PSORTb is a cutting-edge web service that uses various computational techniques to predict subcellular protein locations [19]. Additionally, the AllerTOP 2.0 tool was used to evaluate allergenicity, whereas the Vaxijen 2.0 tool was used to evaluate antigenicity [21, 22]. ProtParam was used to compute stability and molecular weight (MW) [23]. Additionally, the TMHMM 2.0 server was used to forecast transmembrane helices [24]. The TMHMM uses a hidden Markov model to predict membrane protein topology [25]. It excels in predicting transmembrane helices and distinguishing between soluble and membrane proteins [26]. Proteins with high transmembrane helices were removed due to challenges in cloning, purification, and expression that render them unsuitable for vaccine development [24]. The proteins identified as potential vaccine candidates were therefore non-allergenic and antigenic, with no transmembrane helices [18].

### Epitope Prediction and Selection

Predicting T-cell (CTL and HTL) and B-cell epitopes is the key to the epitope selection process [27]. We used MHC-I and MHC-II binding tools from the Immune Epitope Database (IEDB) to predict CTL and HTL epitopes [28]. The protein sequences supplied in FASTA format were subjected to the consensus technique [29]. All alleles were determined to have originated in humans [30]. A consensus score of two was used to determine the epitopes that could bind [28]. ABCPred was used to predict linear B-cell epitopes [31]. These epitopes were subjected to a detailed evaluation using MHC class I immunogenicity, ToxinPred, VaxiJen (v2.0), and Allertop 2.0 servers [32].

### Population Coverage Analysis

To determine the population distribution of the predicted epitopes, we used the IEDB online tool and analyzed the population coverage [33]. This tool provides HLA allele frequencies in 11 different geographic areas across 78 demographic subgroups [34].

### Vaccine Development and Evaluation

When administered as a stand-alone vaccine, epitopes often fail to elicit an immune response because of their limited size [35]. Activation of both the innate and adaptive immune systems requires carriers rich in immunostimulatory adjuvants [36]. In vaccine design, linkers are crucial for mimicking the function of immunogens as distinct immunogenic entities, which helps generate advanced antibody concentrations compared to solitary immunogens [35]. An EAAAK linker was used to attach the cholera enterotoxin subunit B adjuvant to the N-terminal of the vaccine [36]. The EAAAK linker improves the parting of protein domains, facilitating integration of the original and adjuvant CTL epitopes into a bifunctional fusion protein [37]. For the epitope to operate correctly, the two epitopes must be joined [38]. HTL, CTL, and LBL epitopes were linked using AAY, GPGPG, and KK, respectively [38]. AlphaFold2 was used to predict the 3D structure of the vaccine [39]. The vaccination sequence is provided as input for analysis [39]. The GalaxyRefine server was used to further refine the structure [40], which was verified using the PROSAweb, ERRAT, and RAMPAGE servers [41]. Testing for allergens can predict whether a vaccine will cause allergic reactions [42]. Therefore, AllerTOP was used in this study. We investigated structural antigenicity using VaxiJen v2.0 [32]. The ProtParam server was used to assess the stereochemical properties of the vaccine and provide insights into its nature and stability [43]. The Self-Optimized Prediction Method with Alignment (SOPMA) server was used to determine the secondary structure of the vaccine [44]. The Ellipro service was used to predict both conformational and linear B-cell epitopes [45]. The primary input was the three-dimensional structure of the vaccine [45].

### Molecular Docking Analysis

TLR, particularly TLR4, has been shown to recognize bacterial proteins and contribute to the innate immune response against bacterial infections [46]. Therefore, TLR4 (PDB ID: 2Z66) was chosen as the target protein for identifying vaccine candidates [47]. The ClusPro 2.0 server was used to estimate the binding energy between TLR-4 and the vaccine construct [48, 49]. The experiment was conducted by uploading the PDB files of the ligands and receptors to the server and submitting them to default settings [32]. Additionally, a graphical depiction of the interface residues between the vaccine and TLR-4 was obtained using the PDB sum [50]. Using PRODIGY, Gibbs free energy (ΔG) of the vaccine-TLR4 complex was estimated [51]. The detachment constant (Kd) of the vaccine-receptor complex was determined using the formula ΔG = RT × ln(Kd), based on the expected ΔG [52], where R represents the ideal gas constant, and T denotes the temperature in Kelvin [52].

### Molecular Dynamics (MD) Simulations

The methodology used for the MD simulations following protein-protein molecular docking involves several key steps. After identifying the highest-scoring complex, MD simulations were conducted using the GROMACS 2019 package to determine the optimal orientations for interactions between the vaccine candidate and receptor proteins [53]. To create the physiological environment, sodium and chloride ions were added at a neutral salt concentration of 150 mM [54]. The energy of each system was minimized using the steepest descent method until the maximum force (Fmax) was less than 10 kJ/mol, ensuring an energetically favorable initial configuration [53]. The linear constraint solver (LINCS) algorithm was used to maintain constant bond lengths for all the covalent bonds during the simulation. Long-range electrostatic interactions were treated using the particle mesh Ewald (PME) method, with a cutoff radius of 0.9 nm applied for both Coulombic and Van der Waals interactions [55]. Each system underwent two equilibration phases: a 100 ps NVT ensemble to maintain a constant particle number (N), volume (V), and temperature (T), and a 300 ps NPT ensemble to maintain a constant particle number (N), pressure (P), and temperature (T) [56]. These equilibration steps are crucial for stabilizing systems prior to production runs. Simulations were performed under periodic boundary conditions (PBC) in XYZ coordinates to ensure that the atoms remained within the simulation box [57]. Following equilibration, the GROMACS modules were used for subsequent analyses, and visualization and plotting were performed using visual molecular dynamics (VMD) and xmgrace to provide insights into the molecular interactions and dynamics between the vaccine candidate and receptor proteins [58].

### Normal Mode Analysis of MEV Receptor-Docked Complex

The collective motion of the protein vaccine was examined using the iMODS server [59]. NMA was performed using internal coordinates to evaluate the dynamic stability of the TLR-4-vaccine complex [60]. iMODS was used to compute the molecular motion and investigate the structural dynamics of the docking complex [61]. The server organizes the dynamics of the protein complex and provides a range of output data, including B-factors, eigenvalues, covariances, variance maps, atom-by-atom elastic networks, deformability, and residue indices indicating direction and magnitude, among others [59]. The results were uploaded to the iMODS server and analyzed using default parameters with the docked PDB files as input [61].

### Immune Simulation

Immune simulation of the vaccine was performed using the C-IMMSIM server to assess the immunogenicity and immunological response profile of the chimeric peptides [62]. Position-specific scoring matrices (PSSM) created using machine learning approaches are used by the agent-based model C-IMMSIM to predict immunological interactions through peptide prediction [63]. For most currently used vaccines, a minimum interval of four weeks between the first and second doses is recommended [64].

### Reverse Translation, Codon Optimization, and In Silico Cloning of the Vaccine

The vaccine cDNA sequence was produced via codon optimization and reverse translation using the Java Codon Adaptation Tool (JCat), ensuring successful expression of the vaccine in the *E. coli* K-12 strain [65]. The results included a codon adaptation index (CAI) score and GC content, which assisted in estimating protein expression levels [66]. The SnapGene program was used to insert the optimized multi-epitope vaccine sequence into the pET-30a (+) vector, adjusting its orientation and using synonymous codons [67].

## Results

### Proteome Subtraction

In this study, we identified promising candidates for MEV design against *E. corrodens* using a subtractive proteomics technique, in which the less desirable proteins were gradually removed from the proteome. The 2072-protein proteome of *E. corrodens* NCTC 10596 was obtained from UniProt (Accession No. UP000215465). Following the identification of only 393 essential proteins, their resemblance to the human proteome (Taxonomic id: 9606) was scrutinized. Human homologs were removed to stop the immune system from mistakenly identifying its own cells as foreign objects and destroying them. Homologous proteins must be avoided to prevent such circumstances. Of the total, 187 human homologs were detected and subsequently excluded, leaving 206 non-homologous proteins for further analyses. The PSORTb server predicted the locations of the important proteins in the cells; 146 proteins were found to be cytoplasmic, 1 was discovered in the extracellular space, and 41 were located in the membrane region. After removing cytoplasmic proteins, the remaining proteins were chosen for further analysis. Examination of antigenicity revealed that the single extracellular protein was antigenic. Additionally, these proteins have been shown to be non-allergenic, which led to their prioritization as potential vaccination candidates. Immunoinformatics analysis was performed on penicillin-binding proteins.

### Epitope Prediction

HTL, CTL, and LBL cell epitopes were predicted using the vaccine candidates. Of the 29 predicted CTL epitopes, the top 7 were antigenic, non-toxic, immunogenic, and non-allergenic. CTL epitopes were selected for vaccine formulation (Table 1). Similarly, among the 25 predicted HTL epitopes, the top 4 were antigenic, non-toxic, and non-allergenic. HTL epitopes were selected for the vaccine design (Table 2). B-cell epitopes, which are antigenic regions that trigger antibody synthesis, yielded 11 LBL epitopes. However, only the top two antigenic, non-toxic, and non-allergenic LBL epitopes were considered in the multi-epitope vaccine (MEV) design (Table 3).

### World Population Coverage

Geographical areas and ethnic groups have different MHC allele distributions. Therefore, the population coverage must be considered when developing a vaccine. The population coverage of the screened T-cell epitopes was verified. It is expected that 87.83% of the selected epitopes will be present worldwide. Japan had the highest population coverage (96.99%), followed by South America (90.02%), South Korea (90.31%), Southeast Asia (93.06%), and Japan (96.19%). England had the lowest population coverage (77.08%)(Fig. 2).

### Construction of the MEV

The 11 T-cell epitopes (four HTL and seven CTL epitopes) and two LBL epitopes were amalgamated using AAY, KK, and GPGPG linkers to construct the fusion peptide using MEV design.

**Fig. 1:**
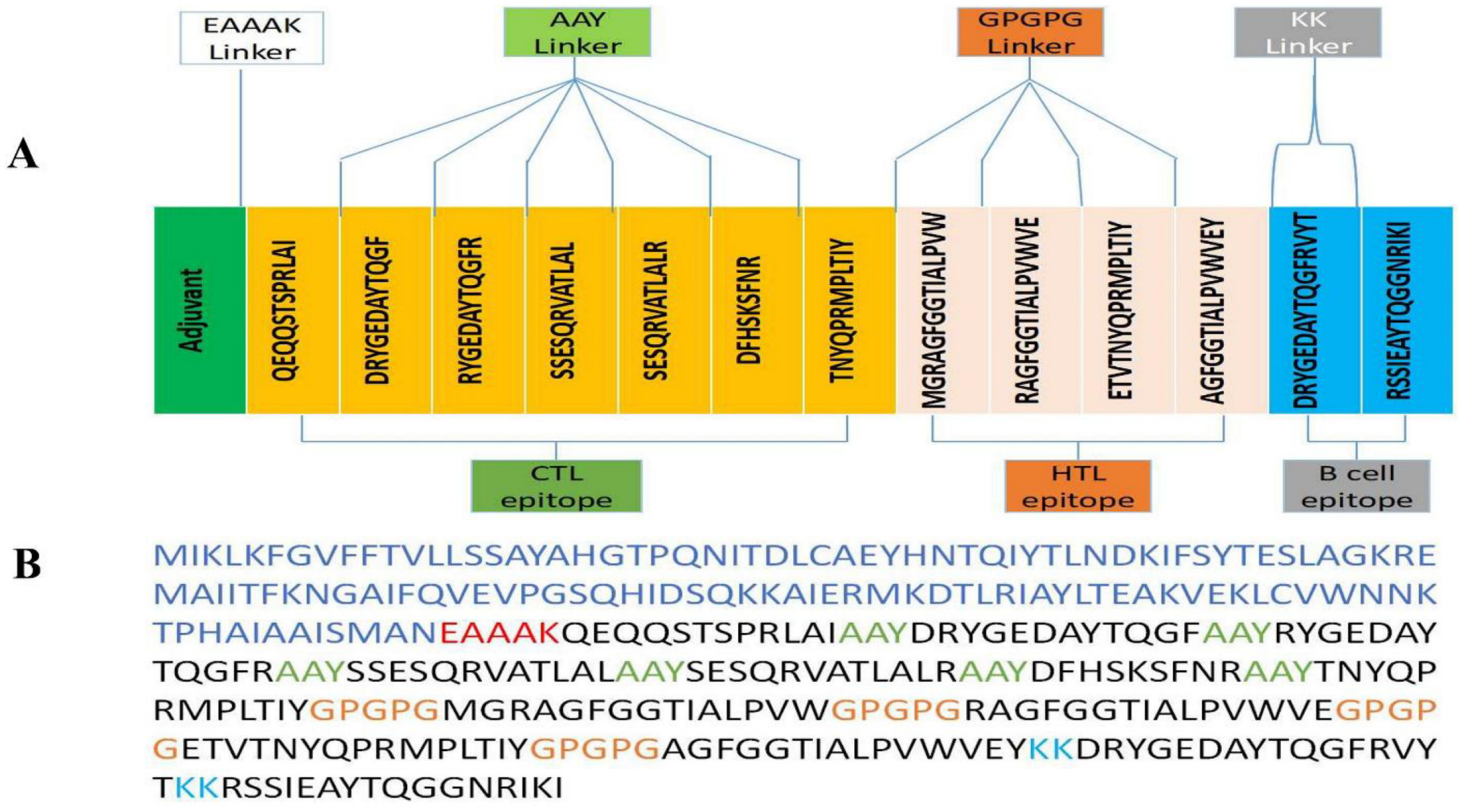
(A) Final MEV construct contain 344 amino acids followed by an adjuvant which is connected by EAAAK linker (purple color) and then followed by AAY connector (grey color) utilized to attach the CTL epitopes & GPGPG connector (cyan color) was utilized to fix the HTL epitopes merged through the KK linker (parrot color). (B) The sequence of the multi-epitope vaccine construct is color-coded: in this case, blue indicates adjuvant, red denotes EAAAK linker, lime suggests AAY linker, blue indicates GPGPG linker, orange shows KK linker, and black indicates CTL, HTL, and B-cell epitopes.

**Fig. 2:**
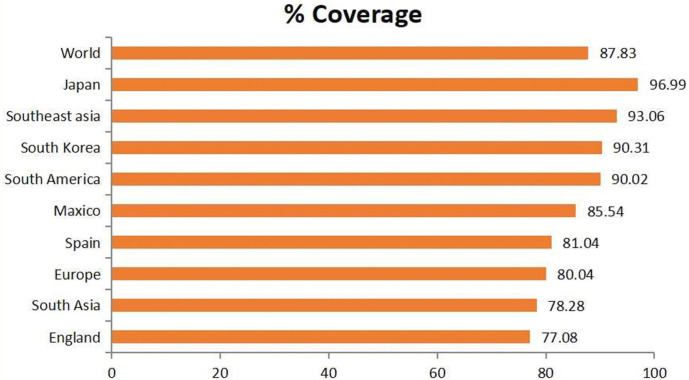
A comprehensive examination of the chosen T cell epitopes' population coverage.

Using an EAAAK linker, the cholera enterotoxin B subunit was fused to the N-terminus of the vaccine to improve antigen-specific immune response. The final construct contained 344 amino acids (Fig. 1).

### Vaccine Construct: A Physicochemical and Structural Study

Following the development of this vaccine, its various immunogenic and physicochemical properties were explored. Upon initial examination, the vaccine did not show homology. Moreover, the construct demonstrated antigenicity with a score of 0.8389 when assessed at a threshold value of 0.5. Further evaluation of toxicity and allergenicity confirmed the non-toxic and non-allergenic nature of the vaccine.

The physicochemical characteristics of the MEV were examined using the ProtParam server. The MEV had a molecular weight of 37.71 kDa and a theoretical isoelectric point (pI) of 9.28; 37 positively charged (Arg + Lys) and 28 negatively charged (Asp + Glu) residues were identified. In addition, the MEV is alkaline because of the greater percentage of positively charged residues and a pI > 7. The MEV was classified as stable with an instability index (II) of 32.68, and thermostability with an aliphatic index of 70.49. The MEV had a hydrophilic score of -0.324 on the GRAVY scale, which is negative. The TMHMM server was used to examine transmembrane helices and we discovered that MEV lacked a transmembrane helix. Collectively, these characteristics indicate that the developed MEV has a strong chance of being approved as a potential vaccine.

The SOPMA tool was used to examine the secondary structure of the MEV. The amino acid sequences of MEVs served as the basis for the predictions produced by SOPMA. Based on the SOPMA data, 141 amino acids (40.99%) form an α-helix, 102 amino acids (29.65%) form coils, and 22 amino acids (6.40%) form β-strands.

Tertiary structure prediction of the MEV was performed using the AlphaFold2 service. The 3D structure of the MEV was further enhanced by 3D refinement, which was used to obtain a total of five structures. Among the pool, Model 5 was selected for additional validation because of its significantly larger Rama-preferred region.

**Fig. 3:**
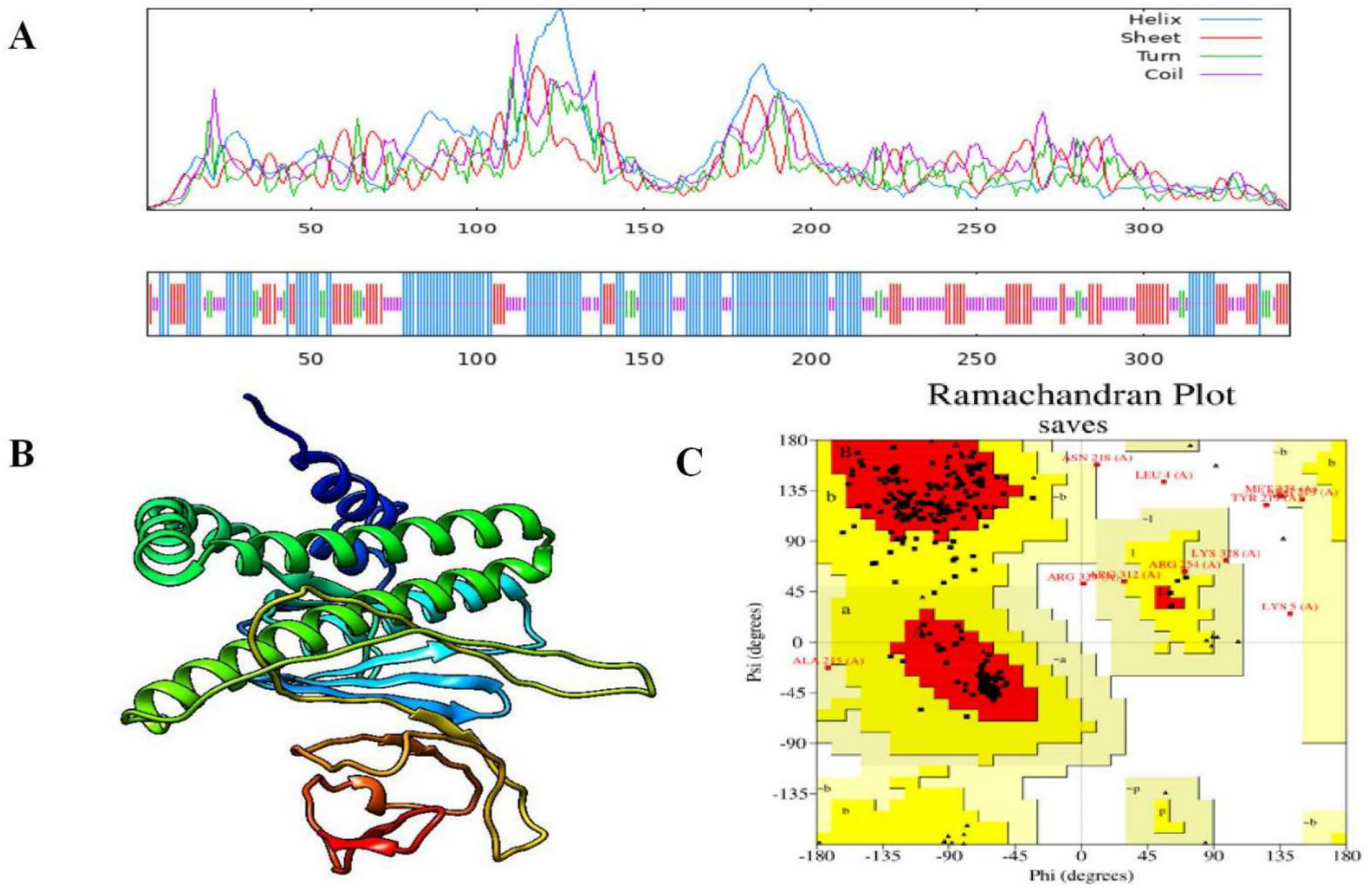
(A) Using the SOPMA technique, the final multi-epitope vaccine construct's secondary structure was predicted (B) The vaccine construct's improved three-dimensional structure (C) 87.2% of amino acids are found in the preferred regions of the vaccine design, according to the Ramachandran Plot, which was created to evaluate its quality.

**Fig. 4:**
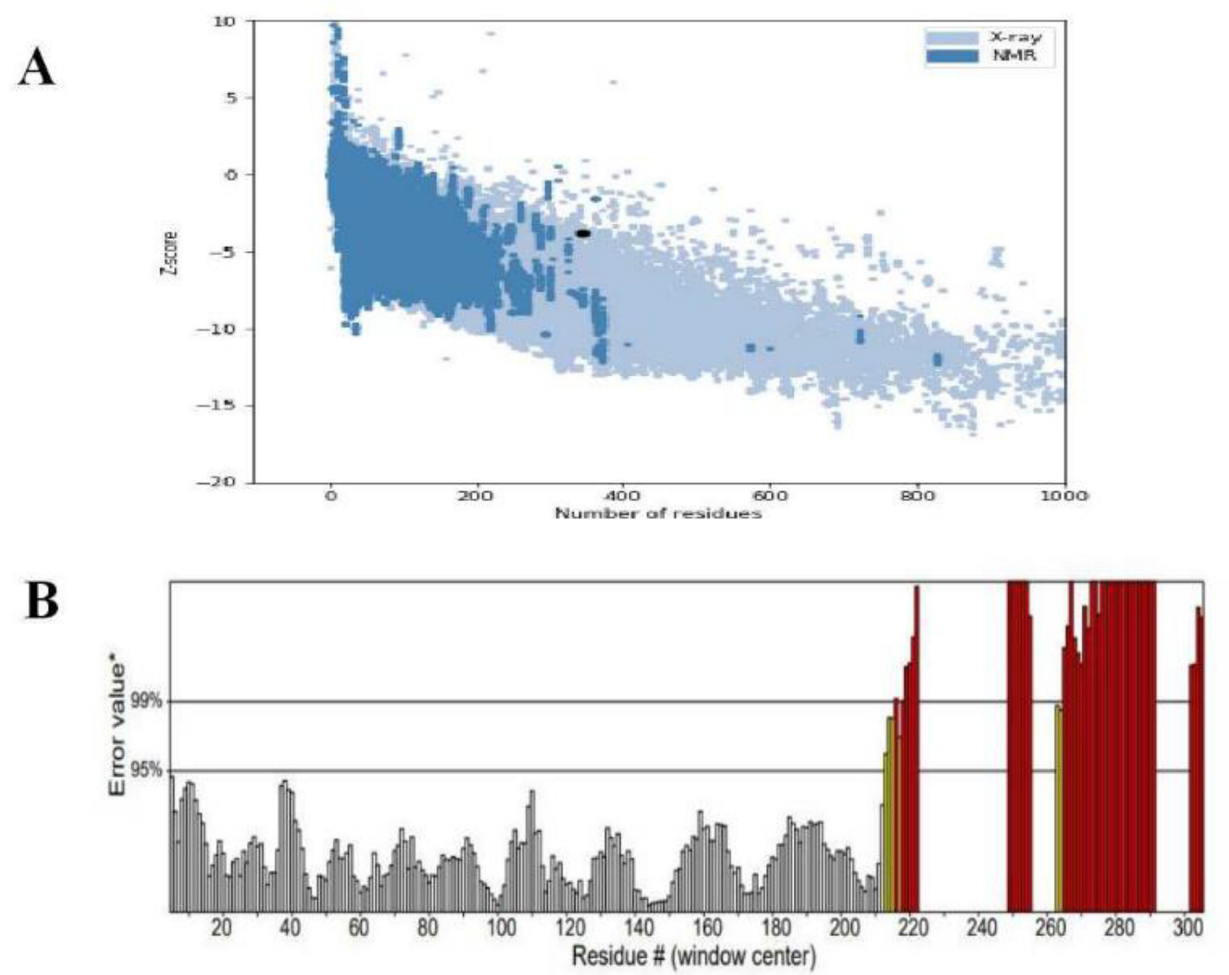
(A) The improved model's Z-score is -3.82 (B) ERRAT score of vaccine construct.

According to the Ramachandran plot produced using SAVES v6.0, 87.2% of the amino acid residues in the vaccine subunit were found in preferred regions, 9.0% in allowed regions, and 1.7% in prohibited regions (Fig. 3). The ProSA web server determined a Z-score of 3.82, which is consistent with high-quality models. Furthermore, the ERRAT analysis yielded an overall quality factor of 76.14, which is higher than the conventional cutoff of 50 for dependable models (Fig. 4).

### Forecasting the B-Cell Epitopes of the MEV

**Fig. 5:**
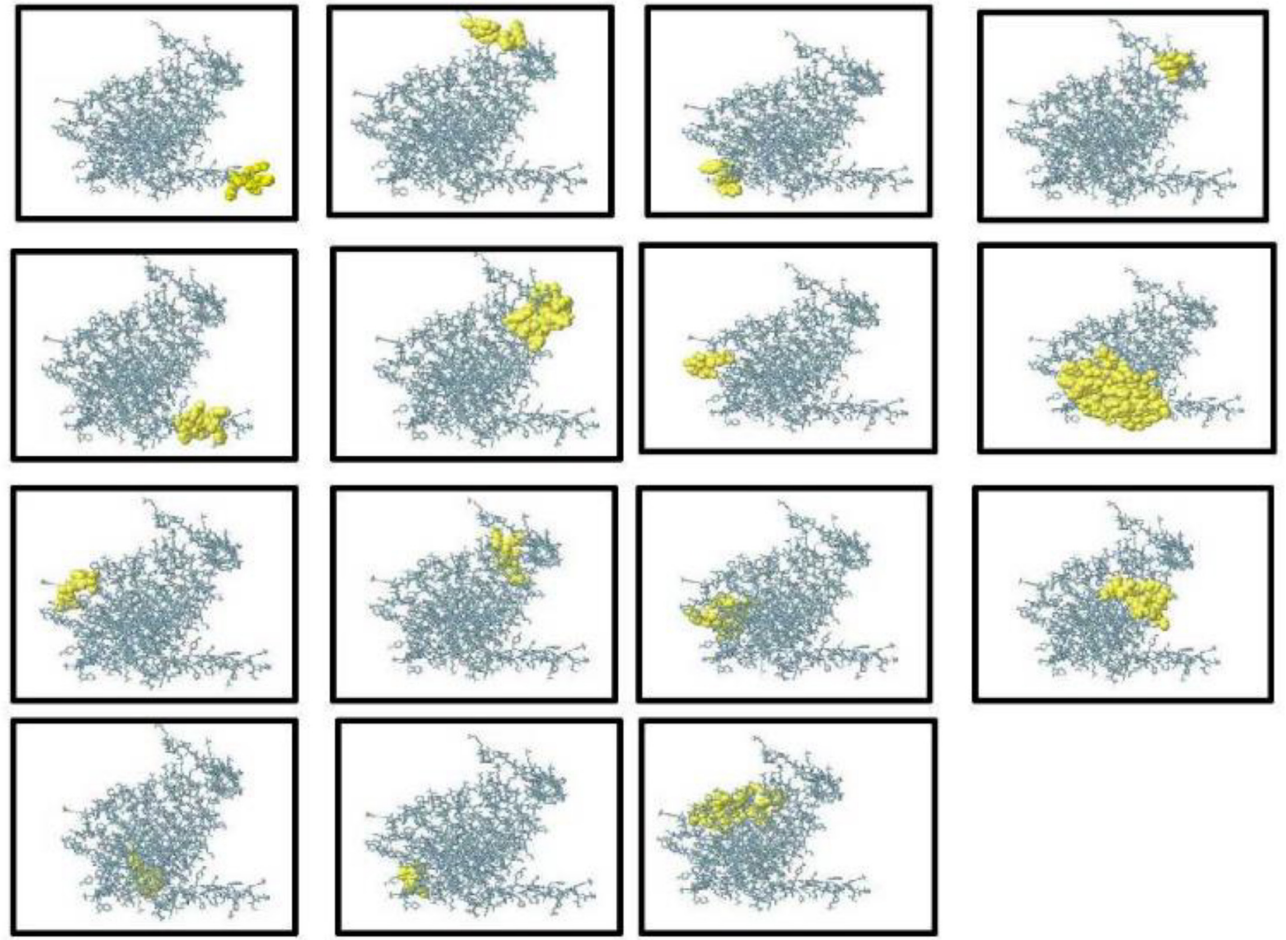
The multi-epitope vaccine contains yellow-colored, conformational/discontinuous B-cell epitopes that are blue in color.

B cells secrete cytokines and produce antibodies that are essential for humoral immunity. As a result, B-cell epitopes must be included in the MEV. The MEV domain was expected to have 12 linear and 15 conformational B-cell epitopes (Fig. 5).

### Interaction of the Vaccine with TLR4

**Fig. 6:**
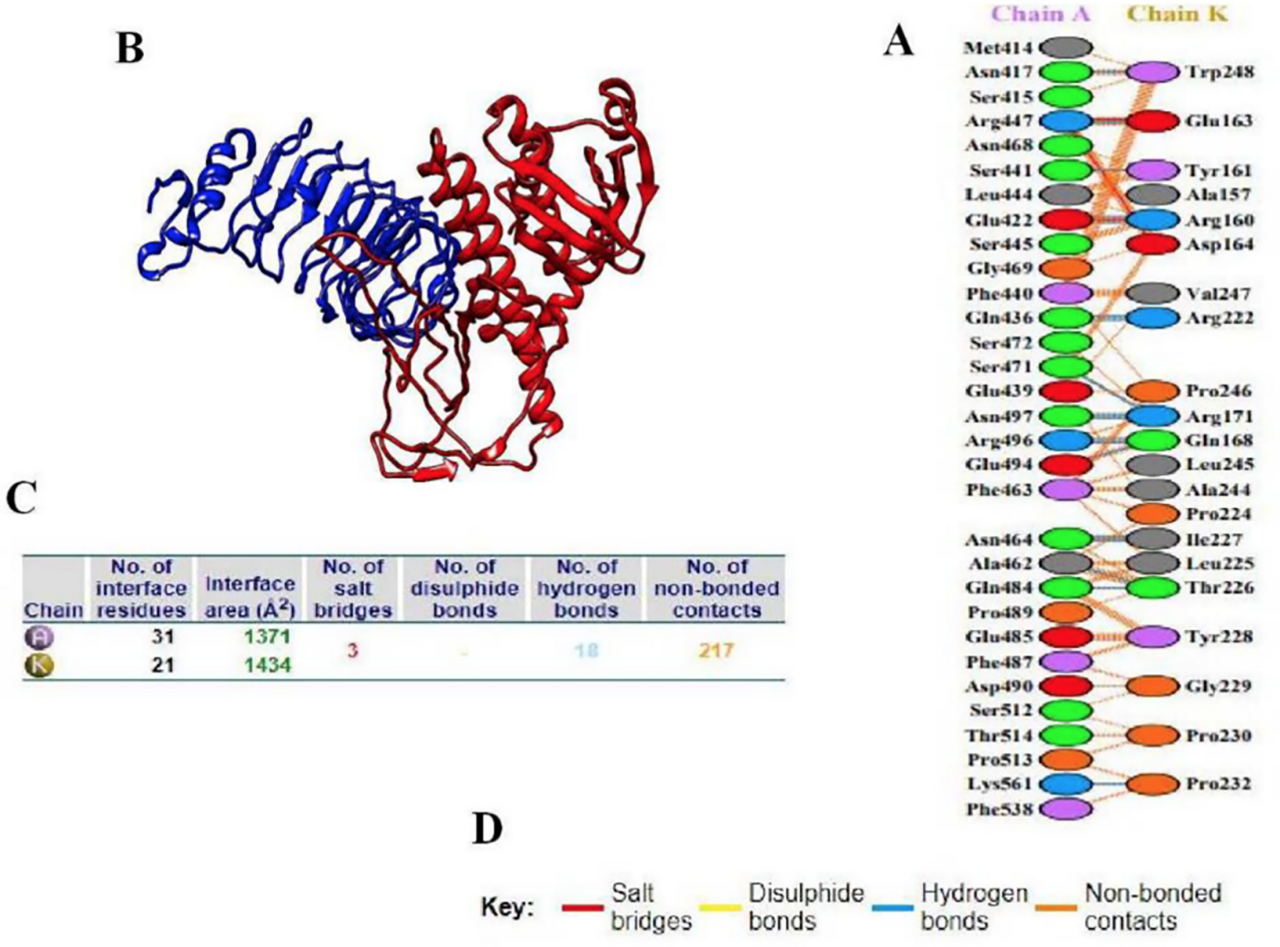
The vaccine component and TLR4 engage in molecular docking. (**A**) Residues that interact with TLR4 (chain A) and the vaccination (chain K). (**B**) The docked complex of TLR4 (blue) and the vaccine construct (red). (**C**) The outcome of interface statistics. (**D**) Key displaying the interactions between residues across the docked molecules' interface.

Protein-protein docking of the designed vaccine and the TLR4 receptor was performed using ClusPro to detect the optimal binding position, evaluate stability, and determine binding affinity. Among the ten generated models, the best docking complex was selected based on two criteria: (1) the highest cluster size and (2) lowest binding energy. The top-ranked model, with 63 members in its cluster and an energy score of -1071.9 kcal/mol, satisfied these criteria, indicating a stable docking complex. PDBsum analysis revealed that the MEV interacted most effectively with chain A of the receptor, forming 18 hydrogen bonds. The thermodynamic parameters of the docking complex were calculated using the PRODIGY web server. At 37°C, the Gibbs free energy (ΔG) was -13.9 kcal/mol, corresponding to an equilibrium dissociation constant (Kd) of 6.4 × 10^-11^, suggesting a strong binding affinity.(Fig. 6)

### Molecular Dynamics Simulation

**Fig. 7:**
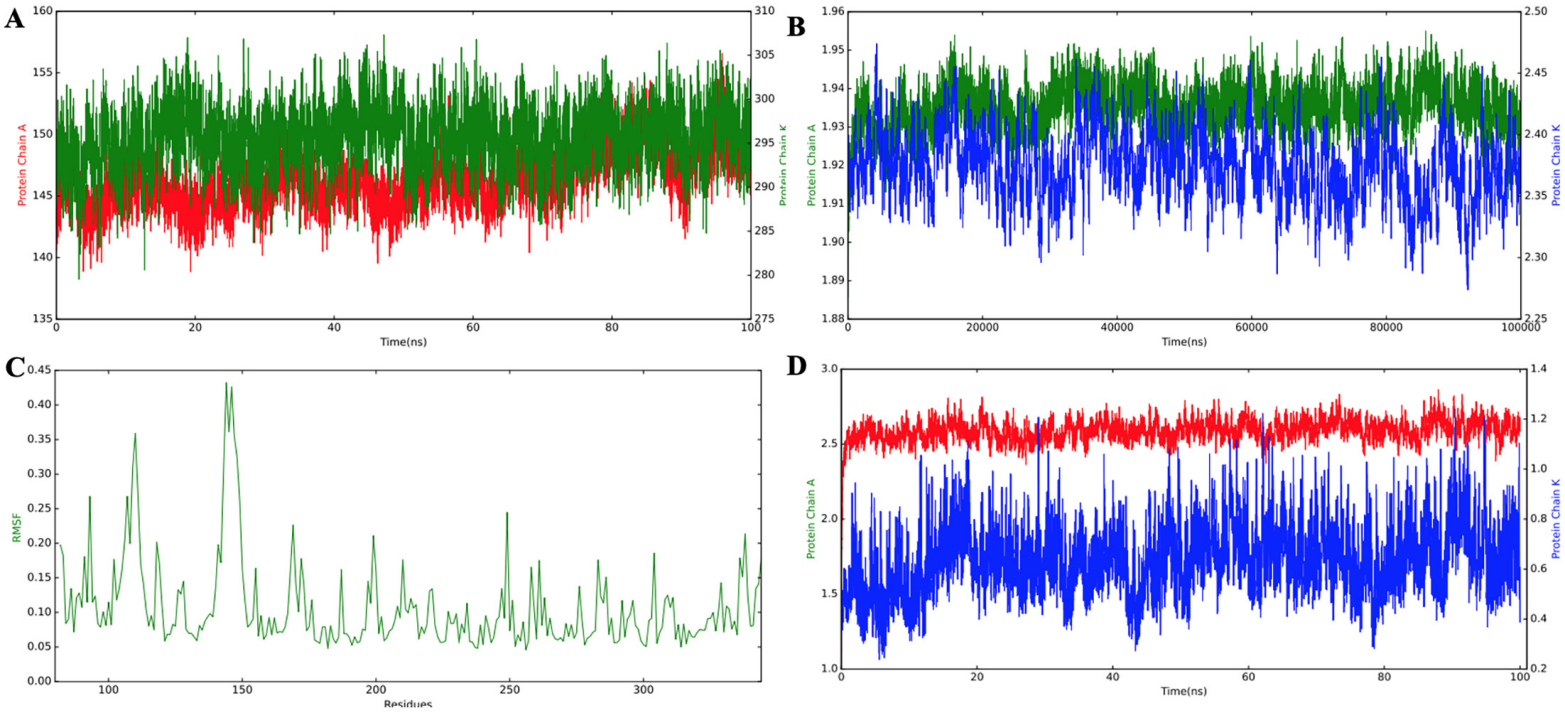
Molecular dynamics simulation of the ligand-receptor complex (vaccine and TLRs). (**A**) The RMSD (Root Mean Square Deviation) analysis indicates the stability of the docked complexes over time. (**B**) The Rg (Radius of Gyration) plot demonstrates that the vaccine construct maintains a stable, compact conformation throughout the simulation period. (**C**) The RMSF (Root Mean Square Fluctuation) plot highlights regions of high flexibility, as indicated by the peaks. (**D**) The SASA (Solvent Accessible Surface Area) analysis provides insights into the surface exposure of the vaccine construct during the simulation.

In this study, we used the GROMACS 2019 software to conduct MD simulations and assess the stability of the interaction between a multi-epitope-based vaccine and the docked TLR-4 complex at a microscopic level. We analyzed the trajectory data using several key metrics: root mean square deviation (RMSD), root mean square fluctuation (RMSF), radius of gyration (Rg), and solvent-accessible surface area (SASA) as functions of time [53]. These analyses provide detailed insights into the binding patterns and conformational variations of the systems under investigation. Complex stiffness and consistency were analyzed using RMSD of the C-alpha atoms of the vaccine-TLR4 docked complex [53]. As illustrated in Fig. 7, the results from the MD simulation indicated that the maximum RMSD values occurred between 20 and 60 ns during the simulation period (Fig. 7A). Furthermore, throughout the entire 100 ns simulation timeframe, the protein exhibited a stable conformation, suggesting robust interaction stability within the complex. While minor oscillations in the plot demonstrate continuous contact between the receptor and ligand molecules, large fluctuations suggest extremely flexible regions in the receptor-ligand complex. In Fig. 7B, the compactness of the protein along its axes is illustrated using an Rg plot. Rg serves as a quantitative measure of the distribution of a protein's atoms around its center of mass, providing insights into structural compactness. The RMSF plot presented in Fig. 7C reveals that the positioning of the amino acid side chains began to fluctuate significantly from the 150 ns mark. This observation suggests ongoing interactions within the protein-receptor complex. Regions exhibiting substantial variations in RMSF values indicate areas of high flexibility, highlighting the dynamic nature of these specific regions within the protein-receptor complex. Such flexibility may play a crucial role in the functional adaptability and responsiveness of the complexes to molecular interactions. Analysis of the total SASA over time illustrated changes in the surface area of the complex. The SASA plot indicates a reduction in the surface area of protein complexes, which correlates with enhanced receptor-ligand interactions [68]. This trend, as depicted in the SASA curve, was inversely related to the RMSD values (Fig. 7D). The results of SASA analysis further reinforced the notion of stable binding between the MEV and TLR4, suggesting that effective interactions lead to a more compact conformation of the complex (Fig. 7).

### Normal Mode Analysis of MEV Receptor-Docked Complex

Using the iMODS online server, normal mode analysis (NMA) was performed to assess the mobility and stability of the docked vaccine-TLR4 complex. The “deformability” of the protein, or its capacity to change conformation in its 3D structure, was the main focus of this investigation.

**Fig. 8:**
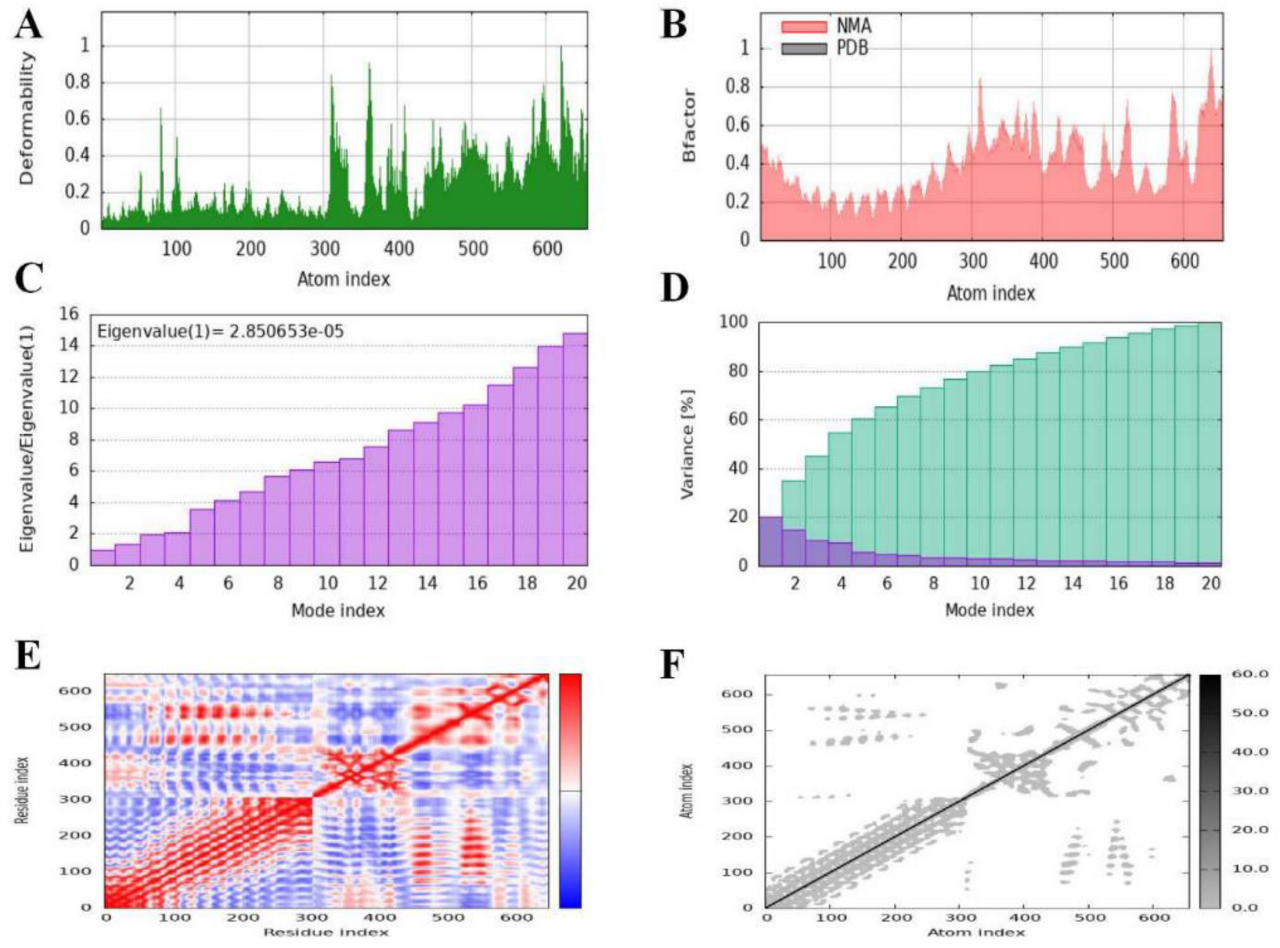
Normal mode analysis of the vaccine protein complex. (**A**) Low main chain deformability was utilized to evaluate the vaccinés stability. (**B**)Mobility or the B factor. (**C**) The protein's normal mode and the stiffness of the motion were revealed by the Eigenvalue. (**D**) Normal mode variance. (**E**) Covariance matrix. (**F**) The elastic network model depicted the residues in a stiffer state.

The graph peaks represent great deformability, *i.e.*, the ease with which the 3D structure of proteins can be altered. The Protein Data Bank (PDB) and NMA of the docked complex were compared using a B-factor graph. The fact that the NMA data in the graph had larger peaks than the PDB data suggests that the NMA data should have predicted higher B-factors. However, the analysis revealed that the B-factor values derived from the experimental PDB data were less indicative of flexibility and mobility than those predicted by computational simulations using NMA. The obtained eigenvalue (2.850653e-05) showed the degree of rigidity of the motion. The eigenvalue of a structure indicates the energy required to deform it. The lower eigenvalues of the graph confirmed the remarkable flexibility and stability of the molecular motion by showing that minimal energy was required to alter the structure. The eigenvalues and variance graphs were inversely related. Individual values are shown in red, and cumulative variation is indicated in green. The covariance matrix illustrates the residue interactions. Correlated movements are shown in red, uncorrelated movements are shown in white, and anti-correlated movements between amino acids are shown in blue (Fig. 8).

### In Silico Cloning

**Fig. 9:**
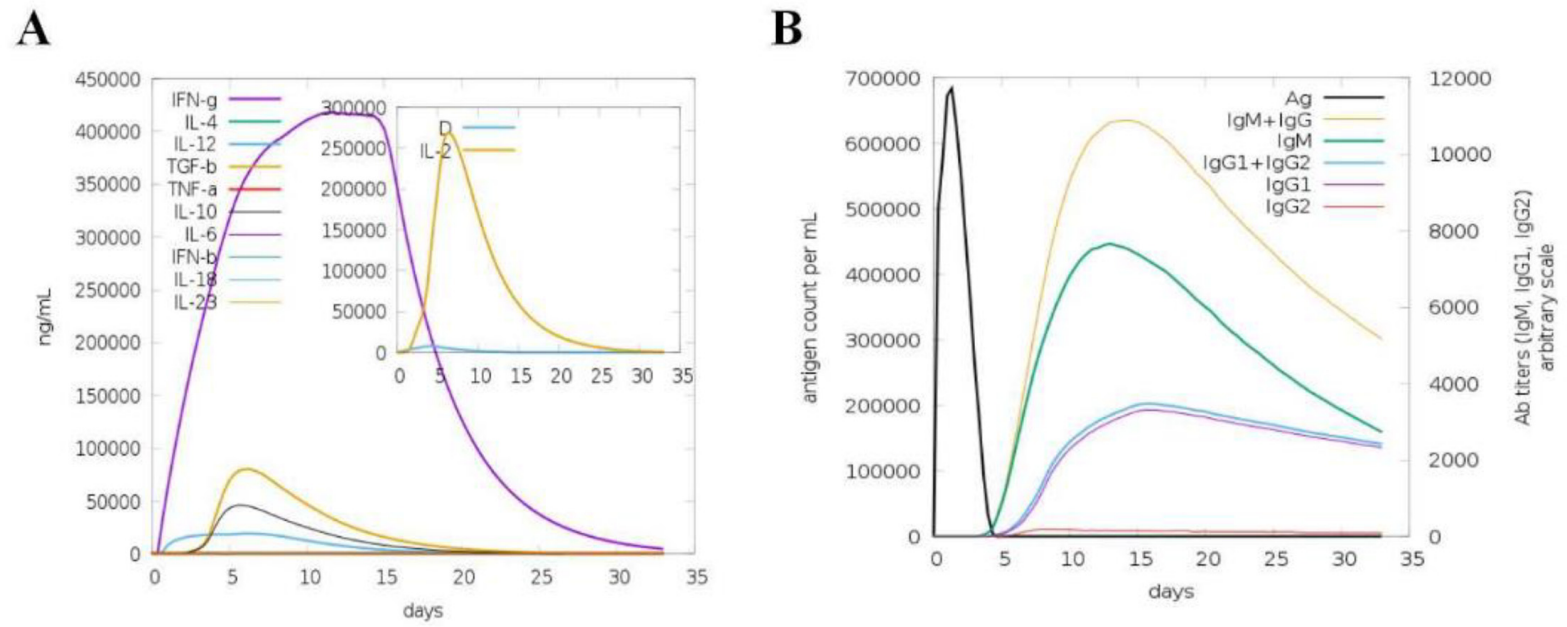
Simulated immunological response where MEV functions as an antigen. (**A**) The production of B-cell isotypes and immunoglobulins in response to antigen exposure; (**B**) cytokine and interleukin production in various stages with the Simson index.

An in silico approach was used to clone the MEV into the pET30a(+) plasmid for expression in an *E. coli* system. Flanking sites were generated by cleaving both the cloned region and the plasmid using NdeI and XhoI restriction enzymes. Prior to this step, the codon used in the construct sequence was changed to correspond to the K12 strain of *E. coli*. Based on the JCAT reports, the optimized sequence had a GC content of 52.6% and a codon adaptation index (CAI) of 1.0. The size of the resulting clone was 5211 bp (Fig. 9).

### Immune Simulation

**Fig. 10:**
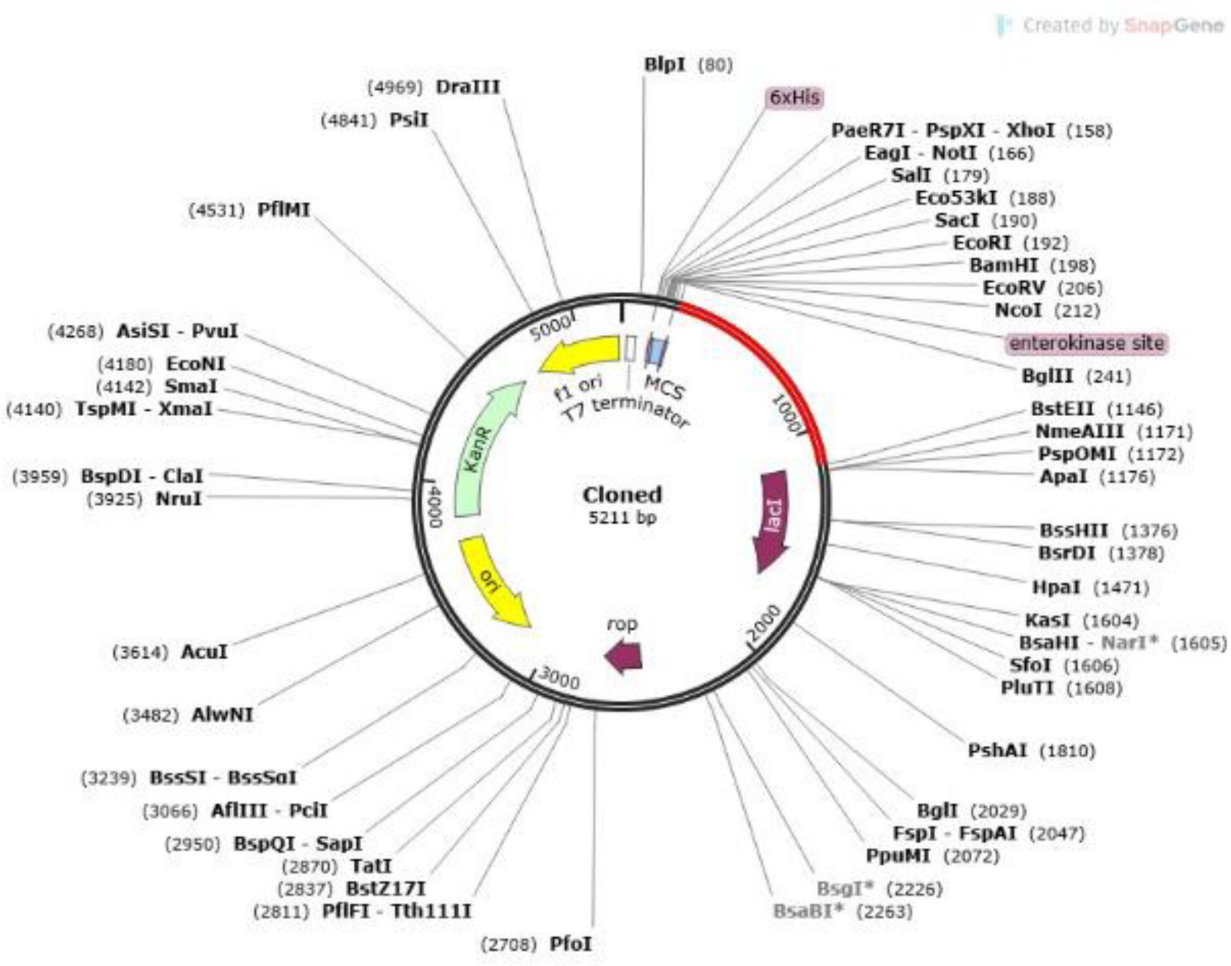
Vaccine cloning *in silico* into the host expression system of *E. coli* K12. The inserted nucleotide sequence is colored red, although the plasmid is shown in black.

The immunogenic profile of the vaccine was assessed using the C-IMMSIM server. All primary, secondary, and tertiary immune responses were observed to play significant roles in establishing immunity through vaccination. Specifically, notable titers of IgG and IgM antibodies were detected, followed by IgG1 and IgM antibodies. Additionally, diverse B-cell isotypes were generated in response to the vaccination regimen, resulting in the formation of memory cells. Furthermore, the vaccine candidate induced increased levels of IL-2 and IFN-γ (Fig. 10).

## Discussion

*E. corrodens* grows slowly and causes serious invasive illness [69]. Although reports of *E. corrodens* infections in many different parts of the human body have been extensively documented, pericarditis caused by invasive *E. corrodens* infection is not common [68, 69]. When purulent pericarditis caused by *E. corrodens* infects immunocompromised individuals, including patients with neutropenia, it progresses quickly and exhibits unusual symptoms [70]. The development of novel treatment approaches is essential because of this pathogen’s potential to cause major health issues. Reverse vaccinology and immunoinformatics are emerging as efficient methods for the quick and affordable identification of new vaccine candidates against lethal diseases [71]. The combination of state-of-the-art technologies and computer tools has greatly simplified the process of discovering and analyzing potential antigens, thereby transforming the field of vaccine development [72]. Our primary aim in this study was to develop an innovative chimeric MEV targeting *E. corrodens*. This vaccine aims to effectively bolster the human immune system, triggering both innate and adaptive immune responses, which offer protection against a range of diseases caused by this bacterium [73].

Our main goal was to identify viable *E. corrodens* vaccine candidates, with a focus on one extracellular protein [74]. The specific protein was selected because of the lack of resemblance to human proteins and their critical role in bacterial survival [53]. This protein is expected to be among the initial molecules that engage with host cells and is predicted to be situated in the extracellular space [60]. As a result, this extracellular protein is an excellent candidate for the development of vaccines since attacking it may induce a strong immune response against *E. corrodens* [75]. In our investigation, penicillin-binding protein 1A (PBP1A) was identified and is considered crucial for the majority of gram-negative bacteria. [76]. PBP1A is involved in the final stages of bacterial cell-wall production [77]. It cross-links the peptidoglycan chains, which are the primary building blocks of the bacterial cell wall [77]. The cell walls of bacteria protect and support them structurally, enabling them to maintain their shape and resist changes in osmotic pressure [78]. Bacterial viability depends on PBP1A, which mediates cell-wall production [79]. Penicillin and other β-lactam antibiotics target PBP1A [80]. However, bacteria can develop resistance to these drugs through various mechanisms, including the synthesis of β-lactamase enzymes, which degrade antibiotics, or alterations to PBPs, reducing the effectiveness of the drug [4]. PBP1A mutations occasionally confer bacterial resistance to β-lactam antibiotics, enabling them to withstand antibiotic treatment [81]. We adhered to stringent protocols for identifying CTL, HTL, and linear B-cell epitopes [82]. B-cell epitopes are necessary for humoral immune responses, which neutralize pathogens and create a memory response for subsequent encounters [83]. T-cells, including both HTLs and CTLs, play vital roles in producing cellular immune responses that stop the spread of illness by either eliminating disease-causing cells or secreting cytokines with antimicrobial properties, thus ensuring long-term protection [84]. Refinement of B- and T-cell epitopes involves additional screening based on antigenicity, lack of allergenicity, and absence of toxicity [85]. Interestingly, the chosen epitopes collectively encompass approximately 87.83% of the global population [86]. These in silico methods have been successful in producing vaccines that offer protection against a range of illnesses [87]. Experimental validation of these in silico vaccine prototypes confirmed their efficacy in eliciting immune responses [88]. It is anticipated that the vaccine models developed in this study will have positive characteristics, such as high antigenicity with low allergenicity and toxicity [60]. The vaccine designs exhibited higher stability and hydrophilicity based on the predicted physicochemical properties [87]. These characteristics suggest that they have the potential to induce robust immunogenic responses in the human immune system [89]. The models also exhibited characteristics such as low molecular weight, thermodynamic stability, and high solubility, suggesting that the vaccine formulations could be efficiently generated and delivered in the host [90].

Understanding the biomolecular interactions between the proposed vaccine and receptor molecules of the human immune cells is crucial for evaluating the effectiveness of immunization [91]. To achieve this, 3D structural data must be obtained. This study validated and predicted the tertiary structures of recommended immunizations using a wide range of computational methods [67]. The quality and stability of the resulting vaccine construction showed significant improvements and expected features, as demonstrated by Ramachandran plots. This validation procedure guarantees that the vaccines under consideration have structural characteristics that facilitate efficient interactions with cellular components of the immune system [59]. Human TLRs are essential for identifying pathogenic peptides and triggering immune responses against certain infections [92]. Molecular docking analyses were performed to assess the efficacy of *E. corrodens* vaccines, with particular emphasis on their interactions with human TLR4 receptors [93]. The purpose of these studies is to shed light on the potential of inducing immune responses against *E. corrodens* by offering insights into the molecular interactions between TLR4 and the proposed vaccine [94]. Stronger immune responses are activated upon repeated exposure to antigenic vaccine formulation [9]. This includes the proliferation of memory B and T cells along with the activation of HTL. Enhanced immunoglobulin production and HTL activation facilitate a robust humoral immune response [95]. Similar immune responses to different infections were observed in a previous study [14]. Moreover, recent investigations experimentally validated predictions produced by the C-ImmSim program [96]. The immunization patterns against the bacterial antigens described in these studies closely matched the predictions generated by C-ImmSim resource [97]. This authentication highlights the consistency of the observed immunological responses under experimental conditions and validates the dependability of the predictions [98]. According to research findings, it is highly likely that the selected vaccine will bind to human TLRs and initiate humoral and cell-mediated immune responses directed against *E. corrodens* [99]. The expression of the vaccine construct in a bacterial expression system was ensured by computational restriction cloning of the vaccine cDNA sequence onto an *E. coli* plasmid [100]. Together, these discoveries lay the groundwork for the development of a vaccine against *E. corrodens* that stimulates strong immune reactions in humans [101].

To address antigenic challenges, the present study introduced a chimeric MEV design integrating components of the PBP1A protein [102]. However, it is crucial to recognize some limitations of the current study. The development of vaccines based on immunoinformatics strongly depends on prediction techniques that introduce uncertainty regarding the degree of protection against PBP1A infections [103]. Standard guidelines, restrictions on prediction techniques, and the availability of high-quality datasets for diverse computer investigations can limit the accuracy of predictive approaches [104]. Although recent case reports have shown promising results from immunoinformatic predictions, the results of this study require verification using in vitro and in vivo bioassays to determine the safety and effectiveness of the suggested vaccine against *E. corrodens* [105].

## Conclusion

This study effectively engaged an inclusive immunoinformatics and subtractive proteomics method to develop an innovative chimeric MEV candidate targeting PBP1A in *E. corrodens*. PBP1A is vital for bacterial growth, which makes it an ideal target for vaccine development. Our designed vaccine construct comprises CTL, HTL, and B-cell epitopes, improved to maximize global population coverage and stimulate both humoral and cell-mediated immune responses. Computational analyses, including immune simulations and molecular docking, indicated that the vaccine established strong binding affinities for human TLRs, along with important immunogenic properties with low toxicity and allergenicity. Moreover, the vaccine construct displayed promising physicochemical properties such as high hydrophilicity, stability, and efficient expression potential in *E. corrodens*. Despite these promising in silico findings, experimental validation, including in vitro and in vivo testing, is imperative to authenticate the safety, efficacy, and potential of the vaccine in effectively combating infections caused by *E. corrodens*.

## Figures and Tables

**Table 1:** Epitopes that were ultimately chosen by CTL to be used in the vaccination against *Eikenella corrodens*.

Epitope	Protein	Allele	Position	Antigenicity	Immunogenicity
QEQQSTSPRLAI	Penicillin-binding protein 1A	HLA-B*44:02	715-726	1.5130	-0.38714
DRYGEDAYTQGF	Penicillin-binding protein 1A	HLAA*24:02 HLA-A*23:01	263-274	1.3572	0.14508
RYGEDAYTQGFR	Penicillin-binding protein 1A	HLAA*24:02 HLAA*23:01 HLA-A*31:01	264-275	1.3115	0.19361
SSESQRVATLAL	Penicillin-binding protein 1A	HLA-B*40:01	281-292	1.2102	-0.06803
SESQRVATLALR	Penicillin-binding protein 1A	HLA-A*68:01	282-293	1.1754	0.01234
DFHSKSFNR	Penicillin-binding protein 1A	HLA-E*01:03	428-436	1.1277	-0.42668
TNYQPRMPLTIY	Penicillin-binding protein 1A	HLAA*24:02 HLA-B*15:02	29-40	1.1163	-0.15988

**Table 2:** Final HTL chosen for the development of an anti-*Eikenella corrodens* vaccine.

Epitope	Protein	Allele	Position	Antigenicity	Immunogenicity
MGRAGFGGTIALPVW	Penicillin-binding protein 1A	HLA-DRB1*07:01	669-683	1.1392	0.47899
RAGFGGTIALPVWVE	Penicillin-binding protein 1A	HLA-DRB1*07:01	671-685	1.1321	0.62862
ETVTNYQPRMPLTIY	Penicillin-binding protein 1A	HLADRB1*08:17 HLA-DRB1*08:13	26-40	0.9851	-0.11236
AGFGGTIALPVWVEY	Penicillin-binding protein 1A	HLADRB1*07:01 HLADRB1*03:09 HLADRB1*03:05 HLADRB1*08:13 HLADRB1*04:08 HLA-DRB1*11:14	269-283	0.9715	0.63105

**Table 3:** The selected B cell to build the vaccine against *Eikenella corrodens*.

Epitope	Protein	Score	Position	Antigenicity	Immunogenicity
DRYGEDAYTQGFRVYT	Penicillin-binding protein 1A	0.93	330	1.3181	0.3399
RSSIEAYTQGGNRIKI	Penicillin-binding protein 1A	0.92	415	1.2933	0.23196
